# The potential impact of COVID-19 on tuberculosis trends in Japan

**DOI:** 10.5365/wpsar.2025.16.4.1169

**Published:** 2025-10-01

**Authors:** Lisa Kawatsu, Kazuhiro Uchimura

**Affiliations:** a Graduate School of Nursing, International Health Nursing, Nagoya City University, Aichi, Japan.; b Research Institute of Tuberculosis, Japan Anti-Tuberculosis Association, Tokyo, Japan.

## Abstract

The COVID-19 pandemic impacted tuberculosis epidemiology worldwide, and Japan was no exception. This report analysed Japan’s national tuberculosis surveillance data to explore the potential impact of COVID-19 on tuberculosis, by age group and place of birth, and to explore possible reasons behind the impact, if any. Overall since 2019, the observed number of notified cases was significantly lower than the number of expected cases. However, closer examination revealed that among Japan-born patients, this was true only for those aged 35–54 years and ≥ 65 years, while among those aged 25–34 years, the observed number of notified cases significantly exceeded the expected cases. Among foreign-born patients, the observed number of notified cases was significantly lower than that of expected cases for those aged 0–24 years and ≥ 65 years. Examination of changes in the modes of detection during the pre- and post-COVID-19 periods revealed that the impact of COVID-19 affected screening opportunities for tuberculosis among various populations differently, which in turn may partially explain the discrepancies between the observed and expected cases among those in different age groups and with different places of birth. A detailed study may be helpful in further understanding the interaction between the impact of COVID-19 on tuberculosis in the short and long-term.

The COVID-19 pandemic has undoubtedly impacted global efforts to control tuberculosis (TB). The World Health Organization has estimated that worldwide in 2020 compared with 2019 there was an 18% reduction in TB case detection, a 15% reduction in the number of people treated for drug-resistant TB, and a 21% decrease in the number of people receiving preventive therapy for latent TB infection. (*1*, *2*) For the first time in more than a decade, TB mortality has increased as well. (*2*) Additionally, it has been shown that the pattern of disruption has varied across regions and countries, probably due to differences in how the COVID-19 pandemic affected TB epidemiology and TB-related health services in local settings. (*3*, *4*)

In Japan, the TB notification rate had gradually been declining, with the annual rate of decline ranging between 4% and 5% since the 1980s; the rate reached 8.2 cases/100 000 population in 2022, with 10 235 newly notified cases. (*5*) Its epidemiology in Japan is unique in that approximately 70% of patients are aged ≥ 65 years, with the greatest number of cases occurring among those aged 85–89 years, followed by those aged 80–84 years. The proportion of foreign-born persons with notified TB is increasing yet still small, accounting for 11.9% of total cases in 2022. (*5*)

COVID-19 was first reported in Japan in January 2020. By the end of April 2023, when the Government of Japan officially downgraded COVID-19 from a class 2 to a class 5 infectious disease (i.e. no longer requiring mandatory notification), a cumulative total of approximately 30 million cases and 70 000 deaths had been recorded. (*6*) Although the age structure among people notified with COVID-19 changed over time – with a greater number of cases being younger people during the latter part of the epidemic – COVID-19 has consistently taken its toll on the elderly: throughout 2021–2023, approximately 85% or more of COVID-19 mortality was reported among those aged ≥ 70 years. (*7*) We thus hypothesized that COVID-19 had affected TB epidemiology unevenly. This report aimed to conduct a detailed examination of the potential impact of the incidence of COVID-19 on the reported incidence of TB, with cases aggregated by age group and place of birth (i.e. Japan-born or foreign-born), and to explore the possible reasons for any impact.

## JAPAN’S TB SURVEILLANCE SYSTEM

Japan introduced its first nationwide computerized TB surveillance system – the Japan TB Surveillance System (JTBS) – in 1987. TB is a notifiable disease, and public health centres are responsible for collecting data about notified patients and entering them into the system. The data are summarized monthly and annually, and selected aggregated data are available online (https://​jata​-ekigaku​.jp/​english) and as annual reports. Mechanisms to ensure data quality include the system’s automatic verification programme, as well as regular training attended by staff from public health centres. (*8*) However, as with any national surveillance system, it is not designed to collect data for research purposes. Thus, some of the information is self-reported, such as country of birth, history of homelessness, HIV status and whether the person has diabetes mellitus, and the JTBS is not linked with any other clinical or health database.

## Methods

Using data from the JTBS, we analysed monthly trends in TB case notification in Japan between 1 January 2017 and 31 December 2022. We explored the potential impact of the COVID-19 pandemic on TB notifications by developing a model to predict the number of expected notifications from January 2020 to December 2022 based on monthly data about reported cases occurring from January 2017 to December 2019. We conducted an augmented Dickey–Fuller test to confirm the stationarity of the time series data, then selected the optimal model from among the autoregressive integral moving average (known as ARIMA), Holt–Winters method, and time series regression models, with the mean absolute percentage error (or MAPE) serving as the evaluation index. Post-prediction analysis involved the evaluation of residuals. Quantile–quantile plots, normality tests, correlation analyses and tests of means between predicted and measured values were conducted to validate the assumptions and performance of the prediction model. We generated predictive models for each age group (0–24, 25–34, 35–44, 45–54, 55–64 and ≥ 65 years) and place of birth (Japan-born, foreign-born and total).

Furthermore, in an attempt to explain the possible reasons behind any changes in the observed and expected trends, we examined the modes of detection for the notified cases. The modes of detection are mandatory data fields and are entered as one of 14 categories, the details of which can be found elsewhere. (*9*) These 14 categories were regrouped into the following, for the purposes of this study: “routine health check” (i.e. a chest X-ray conducted as part of a general health check during entry to university or as part of an annual health check at a workplace or certain social welfare institutions, which are mandatory under the law), “contact investigation” (i.e. for family and casual contacts of active TB cases), “screening at clinical settings with TB symptoms” (i.e. a chest X-ray conducted at a hospital for outpatients presenting with TB symptoms), “screening at clinical settings for other diseases/symptoms” (i.e. a chest X-ray conducted at a hospital for out- and inpatients presenting with other symptoms or seeking care for other disease), and “others and unknown.” The proportions of modes of detection by age group and place of birth for those patients newly notified in 2019 were described, and the percentage change in the number of cases detected in 2019 and 2020 was calculated for mode of detection, age group and place of birth.

All analyses were conducted using R statistical software (version 4.3.2, http://​cran​.r​-project​.org; R Foundation for Statistical Computing, Vienna, Austria).

## Results

Between 2019 and 2020, the notification rate fell from 11.5 to 10.1 cases/100 000 population. The annual rate of decline since 2019 far exceeded that of prior decades, at 10.6%. The number of notified cases also fell, from 14 460 in 2019 to 12 739 in 2020 (**Table 1**). The numbers similarly fell for both Japan-born and foreign-born patients (**Tables 2** and **3**).

**Table 1:** Total no. of newly notified cases of tuberculosis, by age group and year, Japan^a^

Age group (years)	Year					
	2017	2018	2019	2020	2021	2022
**0–24**	**816**	**863**	**798**	**632**	**574**	**474**
**25–34**	**1127**	**1079**	**915**	**882**	**798**	**685**
**35–44**	**1007**	**858**	**837**	**655**	**556**	**479**
**45–54**	**1246**	**1158**	**1093**	**876**	**813**	**666**
**55–64**	**1397**	**1235**	**1115**	**971**	**846**	**742**
** ≥ 65**	**11 196**	**10 397**	**9702**	**8723**	**7932**	**7189**
**Total**	**16 789**	**15 590**	**14 460**	**12 739**	**11 519**	**10 235**

^a^ The total includes Japan-born and foreign-born patients as well as those for whom their country of birth is unknown; the last group is not summarized in the tables because of their small numbers.

**Table 2:** No. of newly notified cases of tuberculosis who were born in Japan, by age group and year, Japan

Age group (years)	Year					
	2017	2018	2019	2020	2021	2022
**0–24**	**289**	**274**	**228**	**189**	**193**	**117**
**25–34**	**531**	**446**	**360**	**331**	**258**	**202**
**35–44**	**747**	**637**	**642**	**435**	**376**	**291**
**45–54**	**1083**	**1001**	**954**	**752**	**664**	**545**
**55–64**	**1274**	**1148**	**1038**	**899**	**760**	**664**
** ≥ 65**	**10 609**	**10 064**	**9345**	**8474**	**7558**	**6854**
**Total**	**14 533**	**13 570**	**12 567**	**11 080**	**9809**	**8673**

**Table 3:** No. of newly notified cases of tuberculosis who were foreign-born, by age group and year, Japan

Age group (years)	Year					
	2017	2018	2019	2020	2021	2022
**0–24**	**514**	**583**	**564**	**437**	**374**	**352**
**25–34**	**565**	**625**	**549**	**546**	**526**	**469**
**35–44**	**219**	**200**	**186**	**214**	**167**	**171**
**45–54**	**114**	**139**	**117**	**110**	**127**	**100**
**55–64**	**65**	**56**	**52**	**54**	**54**	**60**
** ≥ 65**	**53**	**64**	**73**	**50**	**65**	**62**
**Total**	**1530**	**1667**	**1541**	**1411**	**1313**	**1214**

**Fig. 1:**
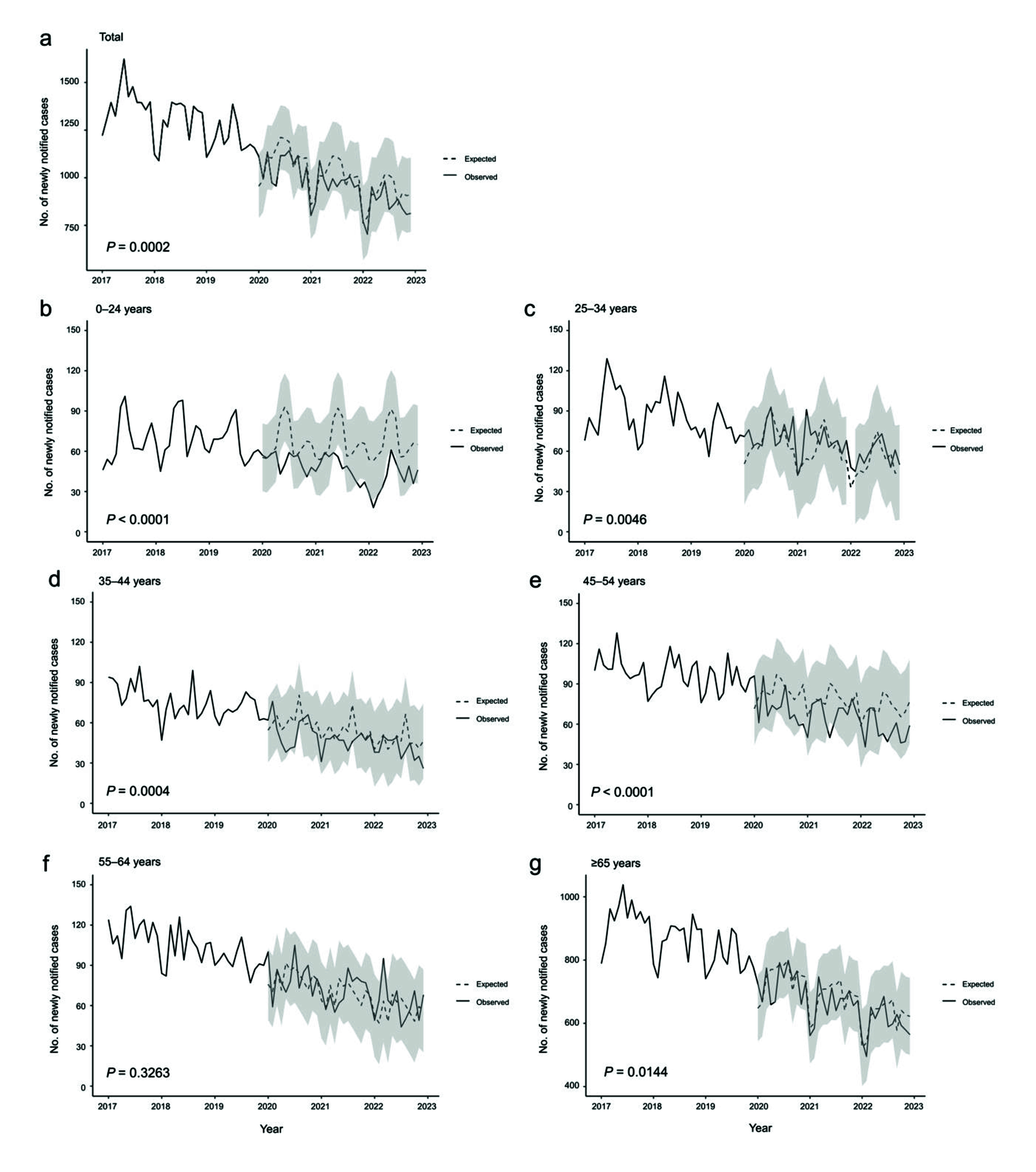
Observed and expected number of monthly notifications of active cases of tuberculosis in Japan, by age group, all patients, 2017–2022

**Fig. 2:**
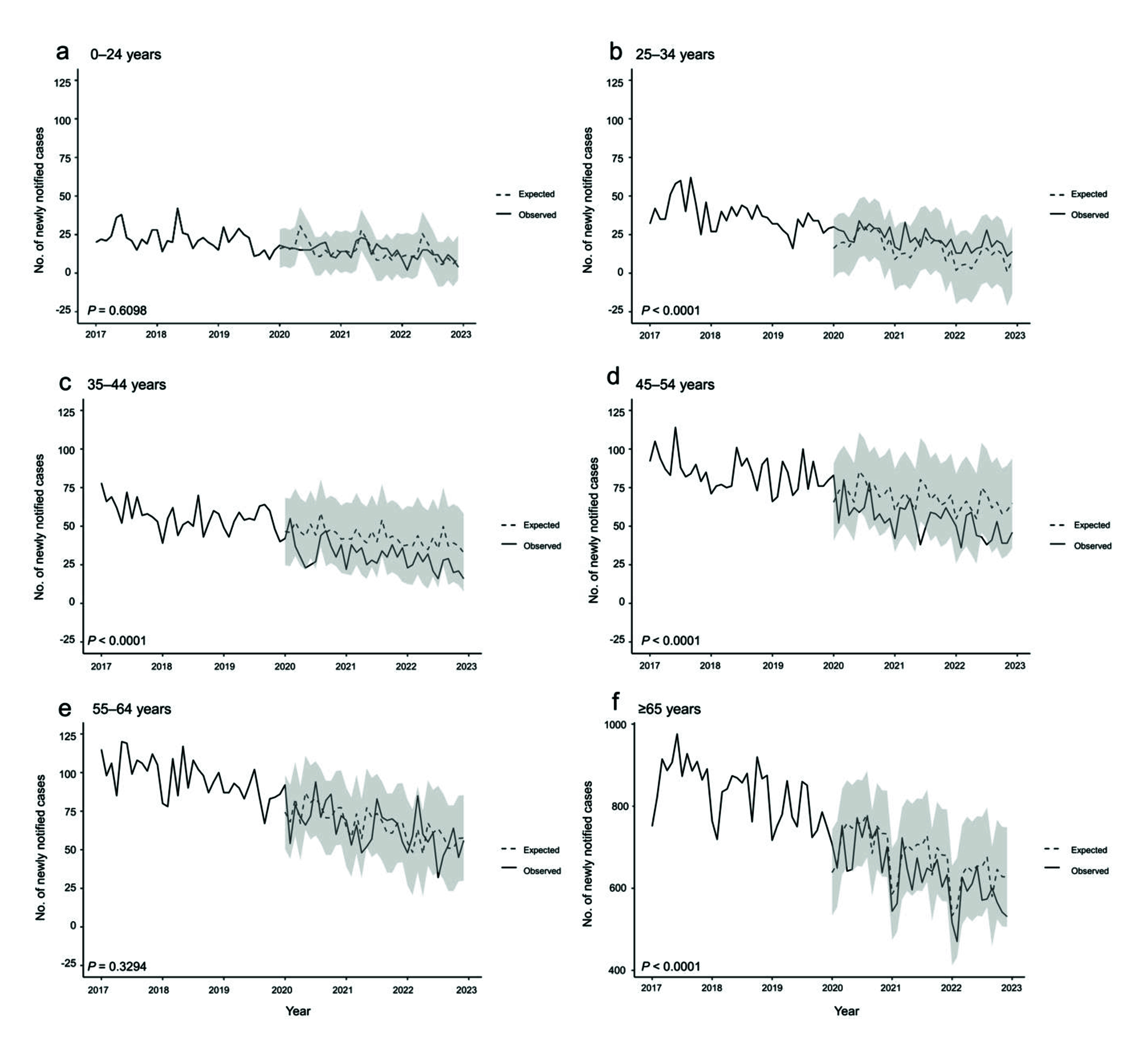
Observed and expected number of monthly notifications of active cases of tuberculosis, Japan-born patients, by age group, 2017–2022

**Fig. 3:**
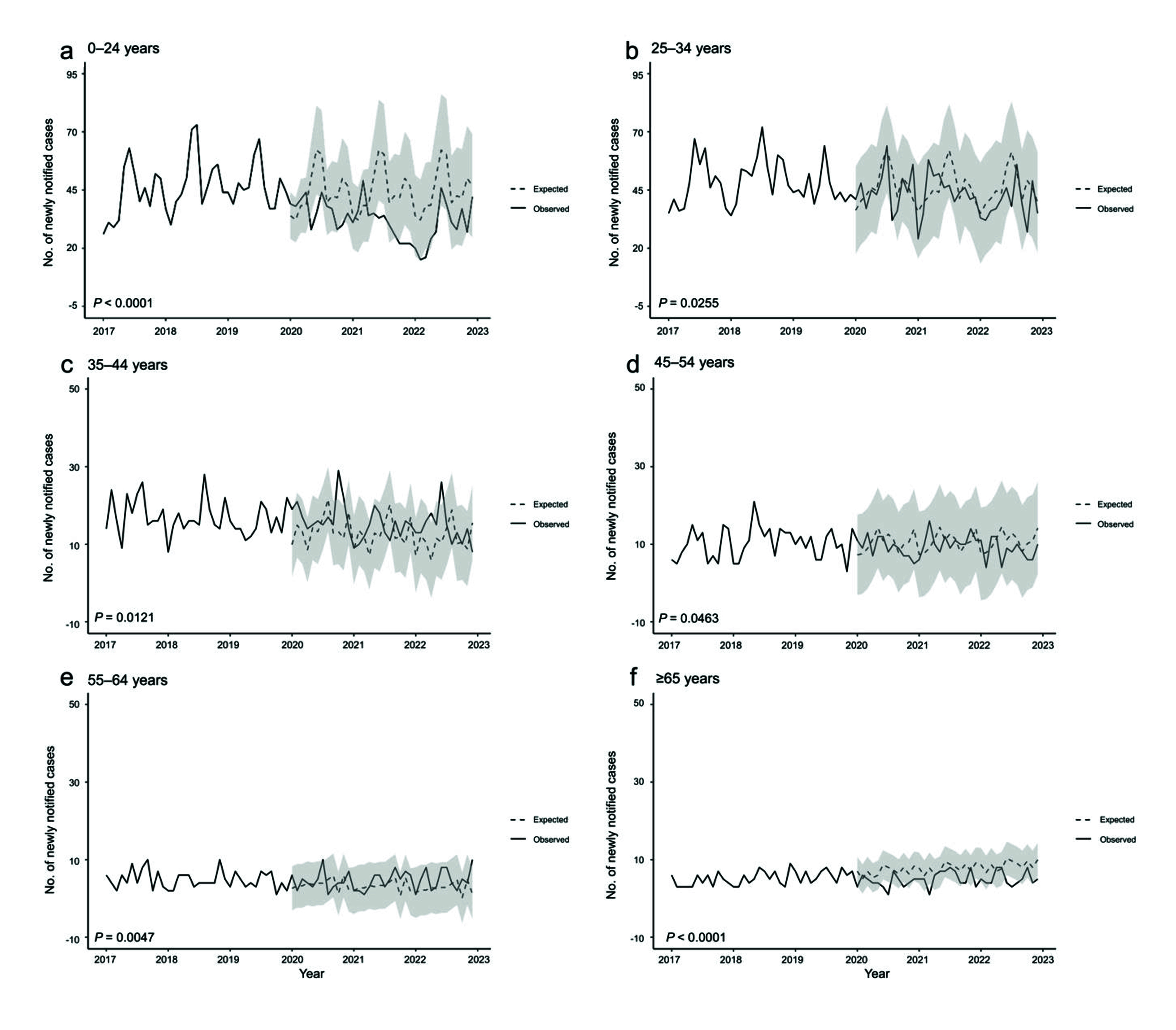
Observed and expected number of monthly notifications of active cases of tuberculosis, foreign-born patients, by age group, 2017–2022

**Fig. 1–3** show the observed and expected numbers of cases by month, from 2017 to 2022, by age group and place of birth. Overall, the observed number of notified cases was significantly lower than the expected number of cases (**Fig. 1**). However, a closer examination revealed that among Japan-born patients, this was true only for those aged 35–54 years and ≥ 65 years, while among those aged 25–34 years, the observed number of notified cases significantly exceeded that of the expected cases (**Fig. 2**). Among foreign-born patients, the observed number of notified cases was significantly lower than that of expected cases for those aged 0–24 years and ≥ 65 years (**Fig. 3**).

[**insert** Figure 1]

[**insert** Figure 2]

[**insert** Figure 3]

**Fig. 4:**
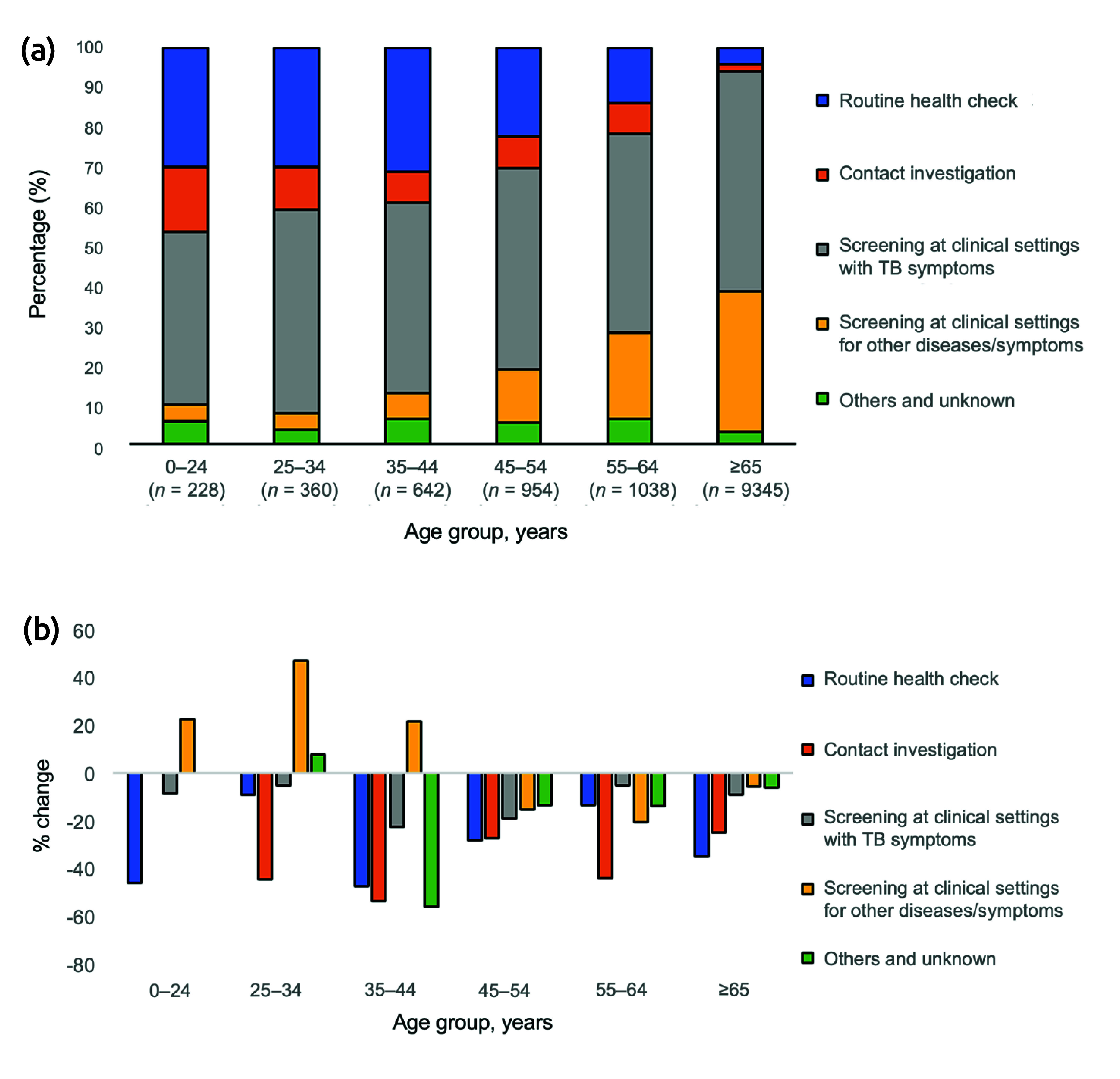
Percentage and change in the numbers of newly notified cases of active tuberculosis (TB), by age group and mode of detection, Japan-born patients (a) Active cases of TB newly notified in 2019, by age group and mode of detection (*N* = 12 567); (b) Percentage change in the numbers of newly notified active cases, by age group and mode of detection, 2019–2020

For both Japan- and foreign-born patients, the main mode of detection was “screening at clinical settings with TB symptoms,” followed by “routine health check” for younger age groups, and for older age groups by “screening at clinical settings for other diseases/symptoms” (**Fig. 4a**, **5a**). Among Japan-born patients, the number of cases detected via “screening at clinical settings with TB symptoms” declined for all age groups, but to varying degrees, with those aged 35–44 years having the largest decline (reduction of 22.9%), followed by those aged 45–54 years (reduction of 19.4%). Furthermore, while approximately 30% of those aged 0–44 years were detected via a “routine health-check,” the number of cases detected via this mode declined significantly for those aged 0–24 and 35–44 years (respective reductions of 46.4% and 47.8%). Conversely, those aged 25–34 years saw only a modest reduction of 9.2%. Furthermore, the number of cases detected via “screening at clinical settings for other diseases/symptoms” increased for those aged 0–44 years, most notably among those aged 25–34 years (increase of 46.7%).

[**insert** Figure 4]

**Fig. 5:**
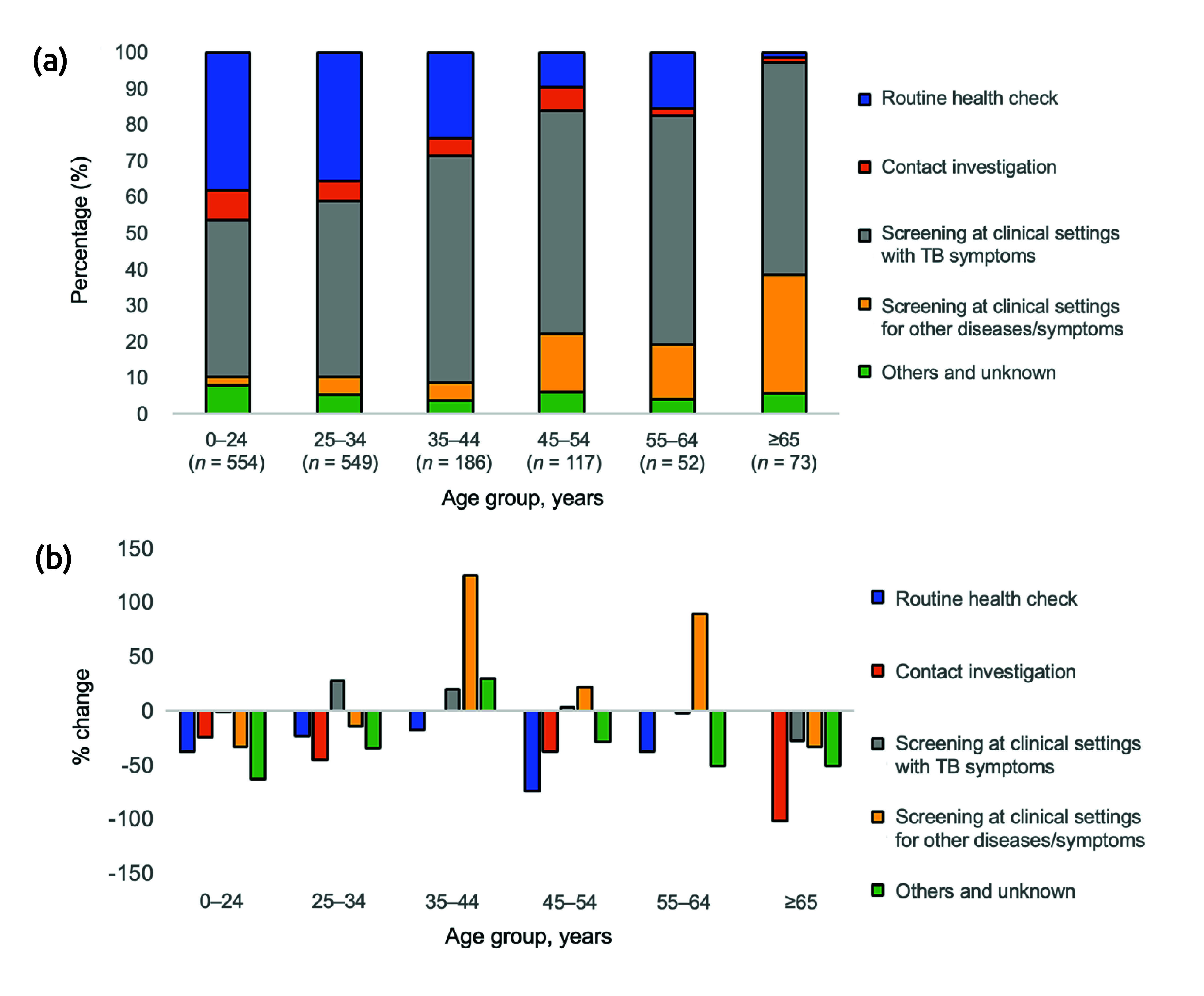
Percentage and change in the numbers of newly notified cases of active tuberculosis (TB), by age group and mode of detection, foreign-born patients (a) Active cases of TB newly notified in 2019, by age group and mode of detection (*N* = 1541); (b) Percentage change in the numbers of newly notified active cases, by age group and mode of detection, 2019–2020

[**insert** Figure 5]

In contrast, among foreign-born patients, the number of cases detected via “screening at clinical settings with TB symptoms” increased for those aged 25–34 and 35–44 years (respective increases of 26.3% and 19.7%), as did the numbers detected via “screening at clinical settings for other diseases/symptoms” for those aged 35–44 years (increase of 122.2%). The number of cases detected via a “routine health check” declined for all age groups.

## Discussion

Both the number of notified TB cases and notification rates declined dramatically between 2019 and 2020, and they continued to do so until the end of 2022. Considering that TB notification did not undergo any systemic changes – that is, there were no changes in the definition of a notifiable case or in contact investigation procedures (*10*) – it is likely that the declines were not system-based. Overall, the observed number of notified cases was below what was expected based on past trends, a phenomenon similarly observed in several other regions and countries. (*11*-*13*) However, there was considerable variation in the relationship between the trends in notified and expected cases by age group and place of birth. Indeed, previous studies have identified a bidirectional impact of the COVID-19 pandemic on TB epidemiology. (*3*, *4*, *14*) Thus, for example, a decrease in community and nosocomial contacts due to lockdown and increased mask-wearing may have directly reduced opportunities for acquiring TB infection, and a decrease in access to TB testing and treatment enrolment due to lockdowns, disruptions in TB services and economic hardship may have reduced TB notifications. (*14*) Alternatively, an increase in household contact, and delays in testing and treatment may have increased the risk of TB infection. (*14*) It has also been suggested that the COVID-19 pandemic may have aggravated poverty and food insecurity, (*15*) which in turn may have increased TB disease burden. Decreases in vaccination against TB with Bacille Calmette–Guérin (*16*) and coverage of preventive therapy have also been reported from Republic of Korea, (*17*) again likely having negative impacts on TB epidemiology.

In Japan, among Japan-born patients, the observed number of notified cases was below what was expected for those aged 35–54 years and those aged ≥ 65 years. This may partially be due to underdetection of TB, especially among elderly people with COVID-19. One study has shown a decline in the number of acid-fast bacteria cultures being submitted to a commercial laboratory. (*18*) However, that study lacked age-specific data, and a detailed study of TB mortality is warranted to further investigate this possibility. Another possible reason for the lower number of notified cases is the decreased opportunities for routine TB screening, as well as suppression of health-care-seeking behaviour and health-care utilization in general. In Japan, annual health check-ups for employees, including chest X-rays, are legally required for most workplaces. For those aged ≥ 65 years, as well as for unemployed and self-employed persons, health check-ups are available and organized by local health authorities. (*19*) However, especially during the early phases of the COVID-19 pandemic, employees were strongly encouraged (but not mandated) to work remotely, thus limiting opportunities for workplace-required health check-ups. (*20*) Furthermore, restrictions on social movement and fear of acquiring COVID-19 from health-care workers, often based on stigma, (*21*) further discouraged people in Japan from accessing health-care facilities. As a result, take-up rates of health check-ups dropped across the country, by as much as approximately 40%, according to one study. (*22*) These observations are also reflected in the decrease in the number of cases detected via the routes of “routine health check” and “screening at clinical settings with TB symptoms.”

It is also worth noting that, unlike other age groups, those aged 25–34 years experienced the minimum reduction in the number of cases detected via “screening at clinical settings with TB symptoms” and the “routine health check” routes, which comprised approximately 70% of modes of detection. The reasons for this are unclear; however, this might explain why the actual number of cases did not decrease as expected. Furthermore, the number of cases detected via “screening at clinical settings for other diseases/symptoms” increased considerably for this age group. This could indicate that TB was detected unexpectedly, as young people who otherwise would not have visited a hospital regularly sought medical care because they became more health-conscious during the COVID-19 pandemic. Indeed, several small-scale studies of students and workers have indicated that people have become more conscious of their diet, exercise and sleep practices. (*23*, *24*) Many people also visited clinics for screening, either simply to reassure themselves before travelling or to receive discounts under the “Go to Travel” campaign, a government-led campaign that provided discounts for travel and leisure activities within Japan in return for people being vaccinated against or tested for COVID-19. (*25*) Another possible explanation is coinfection with TB and COVID-19. Several studies have shown that COVID-19 severity may be worsened by TB. (*26*, *27*) Thus, young people with underlying TB may have had severe COVID-19, encouraging them to seek medical care, during which they were concurrently diagnosed with TB.

The impact the COVID-19 pandemic had on foreign-born cases with TB aged 0–24 years was probably more straightforward. The majority of foreign-born TB patients in Japan are students and workers, including technical trainees from high-burden countries. (*5*) Compared with older age groups, these foreign-born TB patients tend to be diagnosed relatively soon after their arrival in Japan – that is, within 1 to 2 years. (*5*) Hence, border closure measures, which Japan first implemented in April 2020 (and that lasted until restrictions were fully lifted in April 2023, with the number of foreign visitors plummeting by 99.2% at the peak), (*28*, *29*) directly reduced the number of TB notifications from incoming foreign-born persons. However, it is likely that TB among older working-age groups developed several years after migrating to Japan, either as reactivation of a previous infection acquired before arriving in Japan or as an infection newly acquired after migrating to Japan, and thus these groups were less affected by border measures related to the pandemic. This may also partially be reflected in the increase in the number of cases detected via “screening at clinical settings with TB symptoms” and also via “screening at clinical settings for other diseases/symptoms” for those aged 35–44 years. As for those aged ≥ 65 years, due to the small number of cases, it was difficult to ascertain any meaningful changes in the mode of detection between 2019 and 2020.

Our analysis is not without limitations. First, since the JTBS is not designed to collect data for research purposes, some of its data rely on self-reporting and may not be accurate. Second, the JTBS and surveillance data for COVID-19 are not linked; hence, our results are prone to the limitations of an ecological study.

### Conclusion

TB epidemiology in Japan was impacted, both positively and negatively, by the COVID-19 pandemic. Overall, TB notifications fell below what was expected; however, both the direction and the degree of impact differed by age group and place of birth. How the pandemic impacted TB notifications might be explained by complicated interactions between both individual and societal factors. A detailed study, possibly a qualitative study, may be helpful in further understanding the interaction between the COVID-19 pandemic and TB in the short and long-term.
